# Comorbidities of asthma in the elderly in Argentina

**DOI:** 10.1186/1939-4551-8-S1-A111

**Published:** 2015-04-08

**Authors:** Anahi Yanez, Marcela Soria, Susana De Barayazarra, Nancy Recuero, Edgardo Jares, Carlos Bueno

**Affiliations:** 1 Inaer - Investigaciones En Alergia y Enfermedades Respiratorias, Argentina; 2 Hospital Zona General De Agudos Dr. Ricardo Gutierrez, Argentina; 3 Servicio De Alergia e Inmunología Hospital San Roque, Argentina; 4 Private Medical Centers SA, Argentina; 5 Universidad De Buenos Aires, Argentina

## Background

Relatively little attention has been paid to asthma in elderly (AIE) subjects. Our goal is to describe the comorbidities in an Argentinean old population with asthma.

## Methods

An observational descriptive study was performed at five different health care facilities in Buenos Aires. Clinical records during three months of 2014 were searched. Allergists reviewed all clinical histories and elderly was defined as older than 60 years. We evaluated the presence of comorbidities in old patients diagnosed with asthma.

## Results

Total 152 patients were included and their average age (SD) were 66.83 years (6.52), 73% women, 78% Caucasian and 22% Hispanic. Arterial hypertension was the most frequent comorbidity (27%), followed by chronic rhino sinusitis (14%), RGE (10%), diabetes (5%) and obesity (5%). Other comorbidities were also searched, but they were only found in a few patients, such as eosinophilia esophagitis, nasal polyps, dyslipemia, coronary heart disease and pulmonary hypertension. Specifically with respect to allergic comorbidities, patients presented mainly chronic rhino sinusitis (13,8%), and seasonal ( 16%) and perennial rhinitis (16%), or both chronic rhino sinusitis and perennial rhinitis simultaneously (18%). Only a few patients exhibited chronic rhino sinusitis and seasonal rhinitis (0,65%). Considering the well-known association between allergic rhinitis and early-onset asthma (EOA), we observed that most of the patients with EOA exhibited allergic rhinitis (63,4%), but we also observed that half of the patients with long-onset asthma (48%) presented chronic rhino sinusitis or allergic rhinitis.

## Conclusions

Understanding comorbidities associated with AIE may identify at-risk patient populations, improve disease management, and guide treatment advances.

